# Supplementary material for: A high-resolution mRNA expression time course of embryonic development in zebrafish
Source: eLife. 2017 Nov 16;6:e30860. doi: 10.7554/eLife.30860 (PMC5690287; doi:10.7554/eLife.30860)
Supplement: Supplementary file 6. [file elife-30860-supp6.zip › biolayout-clusters-files/Cluster243-genes.html]

Cluster243


# Cluster243: Genes

| Ensembl ID | Gene Name | Chr | Start | End | Biotype |
| --- | --- | --- | --- | --- | --- |
| ENSDARG00000069342 | KLF14 | 2 | 7366943 | 7375242 | protein\_coding |
| ENSDARG00000009346 | caprin1a | 7 | 26727014 | 26750097 | protein\_coding |
| ENSDARG00000018618 | cpsf6 | 4 | 2195819 | 2209989 | protein\_coding |
| ENSDARG00000026180 | prpf8 | 15 | 28316284 | 28329756 | protein\_coding |
| ENSDARG00000077226 | smarca4a | 3 | 19096064 | 19132138 | protein\_coding |
| ENSDARG00000058203 | smc1al | 23 | 13084003 | 13141499 | protein\_coding |
